# Berberine inhibits EGFR signaling and enhances the antitumor effects of EGFR inhibitors in gastric cancer

**DOI:** 10.18632/oncotarget.12589

**Published:** 2016-10-12

**Authors:** Junxiong Wang, Shuo Yang, Xiqiang Cai, Jiaqiang Dong, Zhangqian Chen, Rui Wang, Song Zhang, Haichao Cao, Di Lu, Tong Jin, Yongzhan Nie, Jianyu Hao, Daiming Fan

**Affiliations:** ^1^ Department of Gastroenterology, Beijing Chao-Yang Hospital, Capital Medical University, Beijing 100020, China; ^2^ Department of Laboratory Medicine, Peking University Third Hospital, Beijing 100191, China; ^3^ State Key Laboratory of Cancer Biology and Xijing Hospital of Digestive Diseases, Xijing Hospital, Fourth Military Medical University, Xi'an 710032, China

**Keywords:** gastric cancer, berberine, EGFR, cetuximab, STAT3

## Abstract

Cetuximab plus chemotherapy for advanced gastric cancer (GC) shows an active result in phase 2 trials. Unfortunately, Combination of cetuximab does not provide enough benefit to chemotherapy alone in phase 3 trials. Studies have demonstrated that berberine can suppress the activation of EGFR in tumors. In this study, we evaluated whether berberine could enhance the effects of EGFR-TKIs in GC cell lines and xenograft models. Our data suggest that berberine could effectively enhance the activity of erlotinib and cetuximab *in vitro* and *in vivo*. Berberine was found to inhibit growth in GC cell lines and to induce apoptosis. These effects were linked to inhibition of EGFR signaling activation, including the phosphorylation of STAT3. The expressions of Bcl-xL and Cyclind1 proteins were decreased, whereas the levels of cleavage of poly-ADP ribose polymerase (PARP) were considerably increased in the cell lines in response to berberine treatment. These results suggest a potential role for berberine in the treatment of GC, particularly in combination with EGFR-TKIs therapy. Berberine may be a competent therapeutic agent in GC where it can enhance the effects of EGFR inhibitors.

## INTRODUCTION

Berberine is a natural component derived from Ranunculaceae and Papaveraceae families of plants. Berberine is a commonly used and well-tolerated drug for gastrointestinal disorders [1]. A lot of previous studies have revealed the anti-tumor potential of berberine against various types of human cancer cell lines and xenograft models [2]. In several cancer models, berberine has been proven to stimulate apoptosis, suppress cancer cell growth and arrest cell cycle [3, 4]. Studies also stressed the actions of berberine in inhibiting tumor cell invasion and angiogenesis [5, 6]. The molecular targets of berberine's antitumor activity include p53, AKT, MAPK, STAT3 and NFκB, which can monitor the cell cycle, apoptosis, tumor angiogenesis, and invasion [7]. Berberine also suppresses the activation of some cell growth factor receptors such as EGFR, ERBB2, and VEGF [8].

In spite of the decline in the incidence and mortality of gastric cancer in recent years, the 5-year survival rate of patients with gastric cancer remains relatively low [9]. EGFR, one of the ErbB family of receptors, is overexpressed in gastric cancers, and is related to older age, more aggressive histology and higher disease stage. EGFR expression is correlated with poor clinical outcome in gastric cancer [10, 11]. Cetuximab, an antibody that targets the EGFR, and erlotinib, an EGFR-targeting small molecule tyrosine kinase inhibitor (TKI) are currently under clinical evaluation in gastric cancer trials. However, combination of cetuximab with chemotherapy produced unsatisfactory results for gastric cancer. The PFS and overall survival in the combination therapy are much lower than expected [12].

Berberine-induced inhibition of EGFR has recently been declared in human colon tumor, prostate cancer and human glioblastoma cells [13, 14, 15]. In the current study, we hypothesize that berberine can be used to target EGFR signaling and may enhance the effects of cetuximab or erlotinib.

## RESULTS

### Berberine inhibits cell viability in GC cell lines and decreases the phosphorylation of EGFR

**Figure 1:**
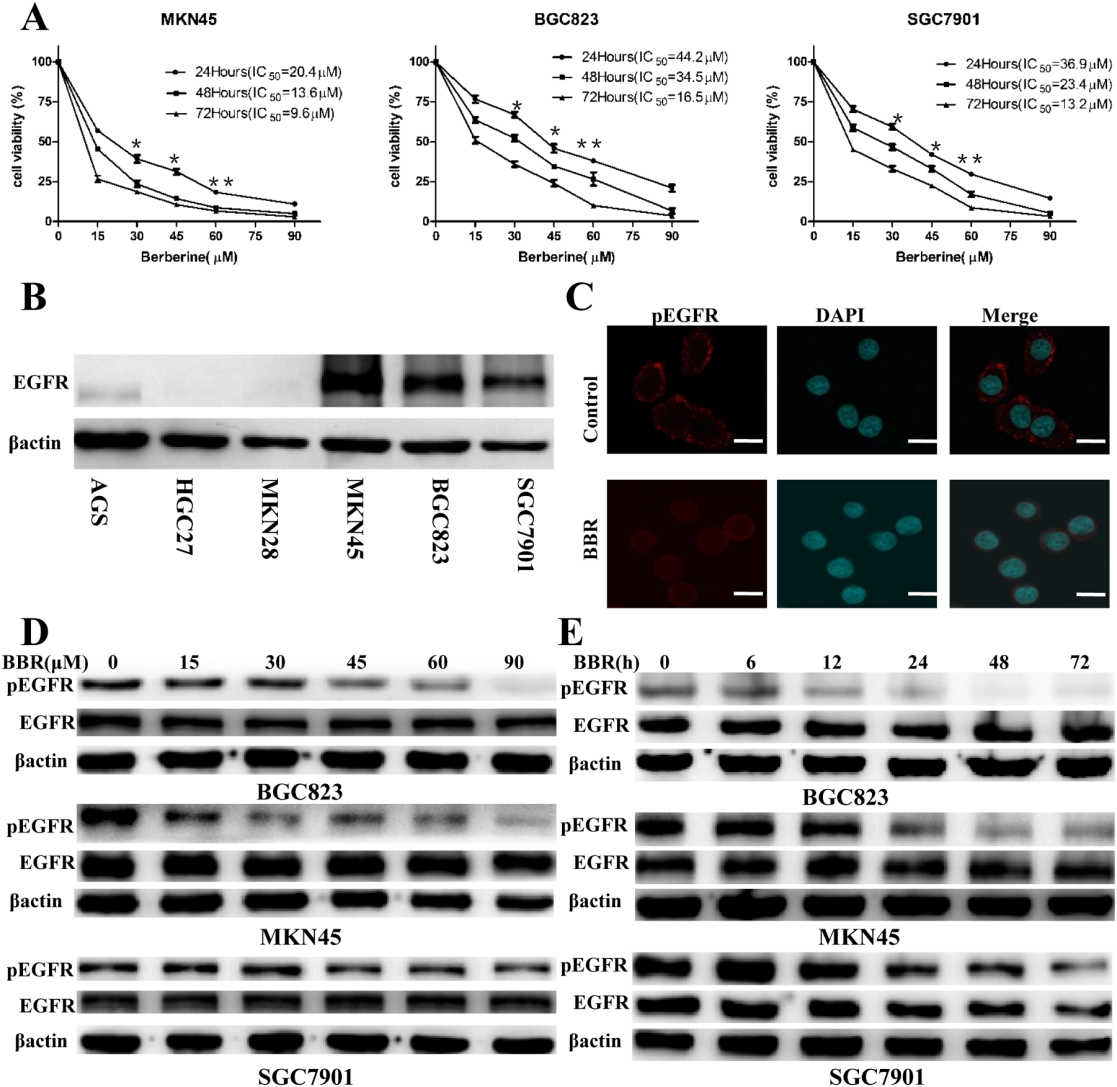
Berberine inhibits cell proliferation and phosphorylation of EGFR in GC cell lines **A.** Cells were treated with the indicated concentrations of berberine for 24 h, 48 h and 72 h, and cell viability was determined by MTT. IC50 values of berberine were shown. * *P* < 0.05 compared with control. ** *P* < 0.01compared with control. **B.** EGFR expression in GC cell lines measured by immunoblot, β-actin was used as a loading control. **C.** Immunofluorescence staining showed that GC cell lines with berberine treatment expressed low levels of phosphorylation of EGFR. The nucleus was stained with 40,6-diamidino-2-phenylindole in the merged images. Scale bars: 30 um. **D.** Cells were treated with different concentrations of berberine for 24 h, and the expression levels of total EGFR and phosphorylation of EGFR were detected by Western blotting. β-actin was used as a loading control. **E.** Cells were treated with berberine (72h IC50) for indicated time. The expression of proteins was evaluated by Western blotting. Representative of three independent experiments was shown. β-actin was used as a loading control. BBR, berberine, DAPI, 40, 6-diamidino-2-phenylindole.

Three GC cell lines, MKN45, BGC823 and SGC7901, were treated for 24,48,72 hours with berberine at concentrations ranging from 15μM to 90μM, and compared to the vehicle (DMSO) alone. Berberine resulted in the decrease of the number of viable MKN45, BGC823 and SGC7901 cells in concentration dependent manners (Figure 1A). MKN45, BGC823 and SGC7901 were EGFR-positive gastric cancer cells (Figure 1B). After 24 hour berberine treatment, we found that phosphorylated EGFR levels decreased and EGFR overall expression levels remained unchanged (Figure 1C, 1D). When gastric cancer cells were treated with berberine(72h IC50) for 72 hours, the level of phosphorylation of EGFR also decline, but EGFR remained unaffected (Figure 1E). It suggests that berberine likely affects EGFR activation.

### Berberine enhances the activity of erlotinib and cetuximab in gastric cells

**Figure 2:**
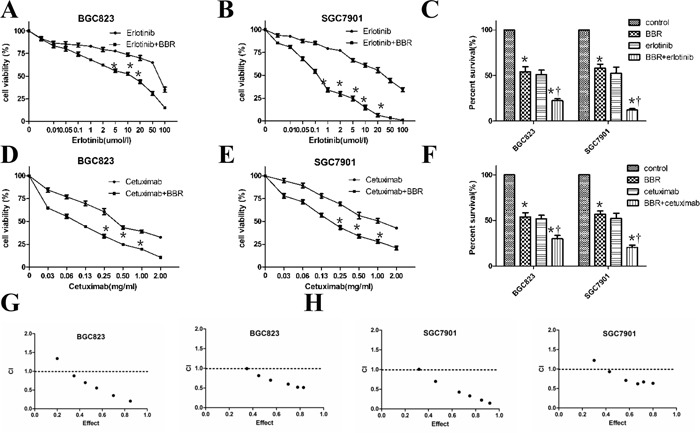
Berberine enhances the activity of erlotinib and cetuximab in gastric cells **A, B.** Berberine increased the sensitivity of SGC7901 and BGC823 cells to erlotinib. Cell viability of cells treated with the indicated doses of erlotinib for 48 hours was assessed with the MTT method. * *P* < 0.05 compared with control. **C.** SGC7901 and BGC823 cells were treated either berberine at its IC50 or erlotinib, both drugs, or their corresponding vehicles. After 48hours, cells were tested with the MTT method. The experiment was performed 4 times with triplicate samples and similar results. * *P*<0.05 compared with control, †, *P* < 0.05 compared with berberine treatment. **D, E.** SGC7901and BGC823 cells were treated either berberine or plus cetuximab with the indicated doses for 48 hours assessed with the MTT method. * *P* < 0.05 compared with control. **F.** SGC7901and BGC823 cells were treated with berberine at its IC50 or cetuximab, both drugs, or their corresponding vehicles. After 48hours, cells were assessed with the MTT method. * *P*<0.05 compared with control, †, *P* < 0.05 compared with berberine treatment. **G.** BGC823 cell was treated with berberine and erlotinib or cetuximab. The combination index (CI) was calculated by median dose analysis. CI smaller than one indicated synergism between two drugs. **H.** SGC7901 cells was treated with berberine and erlotinib or cetuximab The combination index (CI) was calculated by median dose analysis. CI smaller than one indicated synergism between two drugs.

Berberine was tested for its ability to enhance the antitumor effects of EGFR inhibitors in gastric cancer. We used erlotinib and cetuximab in SGC7901, BGC823 cell culture experiments by MTT assays. Berberine enhanced the growth inhibition seen with erlotinib (Figure 2A, 2B, 2C) or cetuximab (Figure 2D, 2E, 2F) treatment *in vitro*. Combining berberine at its IC50 in treatment of SGC7901 cells (48 μM) with the approximate IC50 for erlotinib (30 μM) resulted in a 80.5% growth inhibition, compared to 52% growth inhibition for erlotinib alone, a 1.5-fold enhancement, The median effect analysis showed that the combination index (CI) was smaller than 1 (Figure 2G, 2H), indicating the synergism between berberine and cetuximab or erlotinib.

### Berberine and erlotinib synergistically enhanced apoptosis and cell cycle arrest in gastric cells

**Figure 3:**
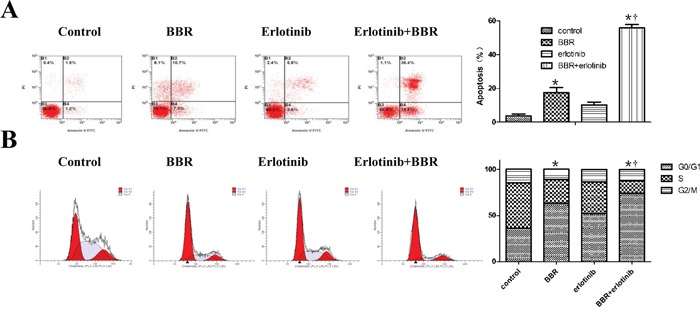
Berberine and erlotinib synergistically enhanced apoptosis and cell cycle arrest in gastric cells **A.** Berberine (30umol/L) and erlotinib (IC2548h) synergistically enhanced the apoptosis of BGC823 cells, Cells were staining with FITC-conjugated Annexin V antibody and propidium iodide (PI) staining for flow cytometry. * *P* < 0.01compared with control, † *P* < 0.01 compared with berberine alone. **B.** Berberine in combination with erlotinib induces cell cycle arrest in gastric cancer cells. Cells were treated with berberine at 30umol/L in the presence or absence of erlotinib (IC2548h) treatment for 24 hours. Percentages of cells in G1/G0, S, and G2/M phase were shown measured by FACS analysis. Images are representative of 3 independent experiments. * *P* < 0.01compared with control, † *P* < 0.05 compared with berberine alone. BBR, berberine.

We next analyzed the induction of apoptosis and cell cycle in BGC823 cells treated with berberine alone or in combination with erlotinib. Flow cytometric analysis revealed that berberine alone induced the apoptosis and cell cycle arrest of BGC823 cells, and the combination therapy further augmented this effect (Figure 3A, 3B).

Taken together, these *in vitro* data suggest that the combined use of berberine enhances the activity of erlotinib and cetuximab in gastric cancer cells.

### Berberine inhibits EGFR signaling pathway

**Figure 4:**
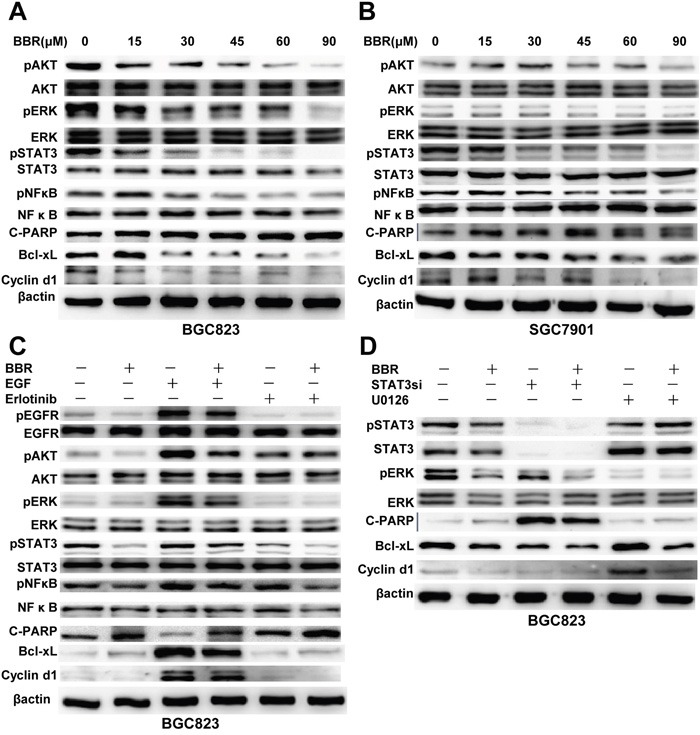
Berberine inhibits the EGFR signaling pathway in GC cells **A, B.** Cells were treated with different doses of BBR for 24 h. Whole cell lysates were probed for pAKT, AKT, pERK, ERK, pSTAT3, STAT3, pNFκB, NFκB, Bcl-xL, cyclin D1, C-PARP and with β-actin as a loading control. Each experiment was performed 3 times with similar results. **C.** Berberine decreased EGFR signaling. Whole cell protein lysates from cells with different treatments were immunoblotted with antibodies as indicated, andβ-actin was used to confirm equal gel loading. Similar results were obtained in 3 independent experiments. **D.** The effect of STAT3 knockdown and selective inhibition of ERK on berberine induced EGFR degradation. Cells were treated with STAT3 siRNA or U0126 and then with berberine for 24 hours. Similar results were obtained in 3 independent experiments. BBR, berberine.

Berberine inhibits EGFR downstream molecules such as:STAT3, AKT, ERK, NFκB, as well as declines in expression of Bcl-xL and cyclinD1, which regulate apoptosis and cell cycle, respectively (Figure 4A, 4B). These data indicate that by inhibiting both EGFR and downstream AKT, ERK, STAT3 activation, berberine may have potential utility in the treatment of gastric cancer.

We detected that berberine inhibited EGFR signaling pathway and downstream targets Bcl-xL and cyclinD1. Then this inhibitory effect of berberine was weakened when cells were pretreated with EGF. Combining berberine with erlotinib, inhibitory effect was amplified (Figure 4C). Furthermore, siRNAs for STAT3 were synthesized and their inhibitory effects on STAT3 were confirmed by western blotting. Remarkably, silencing of STAT3 in BGC823 cells decreased Bcl-xL and cyclinD1 protein expression, and also increased apoptosis marker cleaved PARP (Figure 4D).

These data suggested that berberine inhibited the phosphorylation of EGFR may regulate Bcl-xL and cyclinD1expression via STAT3.

### Berberine enhances the growth inhibitory activity of cetuximab and inhibits EGFR signaling *in vivo*

**Figure 5:**
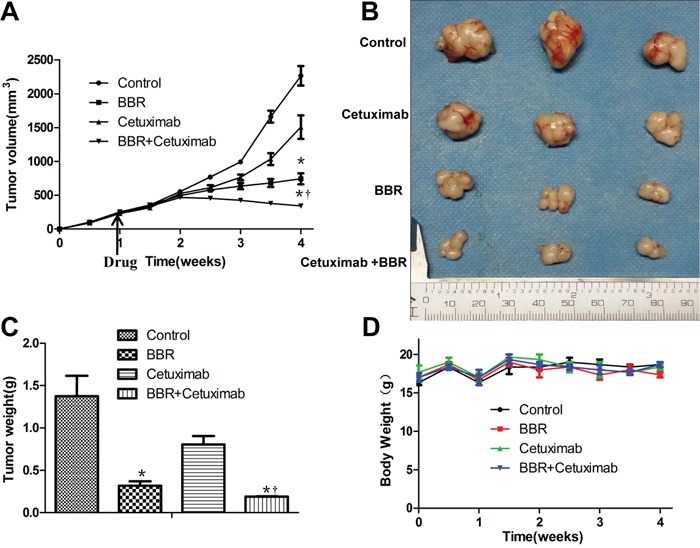
Berberine together with cetuximab suppresses tumor growth *in vivo* **A.** When tumors reached 100 mm^3^ in about 1week, drug treatment started. Tumor volume (mm3) of BGC823 cells treated with cetuximab, berberine, and their combination, *P < 0.01 when compared with the control; † *P* < 0.05 as compared with berberine alone. The time of treatment starting should be indicated. **B.** Macroscopic appearance of the tumors at 4 weeks after drug administration. **C.** Weight of tumor samples from nude mice. * *P* < 0.01 when compared with the control; † *P* < 0.05 as compared with berberine alone. **D.** Body weight of xenografts. No obvious difference was detected in the body weight of mice among the four groups BBR, berberine.

In order to assess whether berberine could enhance the anticancer activity of EGFR inhibitors *in vivo*, as was seen *in vitro*, female nude mice were inoculated with BGC823 cells. After tumor outgrowth and randomization to treatment groups, mice were treated with vehicle, cetuximab alone, berberine alone, or berberine plus cetuximab on alternating days. Berberine was found to significantly enhance the growth inhibitory activity of cetuximab. Treatment with cetuximab slightly slowed down the tumor growth in xenografts. Berberine alone resulted in xenograft shrinkage significantly. Importantly, combination berberine with cetuximab further reduced the tumor size in this model. After 4 weeks the animals were sacrificed, the combination therapy caused a 66% decrease in tumor volume as compared with the control group, whereas cetuximab alone and berberine alone caused a 32%, 55% decrease respectively (Figure 5A). Similarly, the combination therapy showed significantly decreased tumor weight (Figure 5B and 5C). During the experiments, no obvious weight loss was observed in mice treated with berberine, cetuximab, or both (Figure 5D).

**Figure 6:**
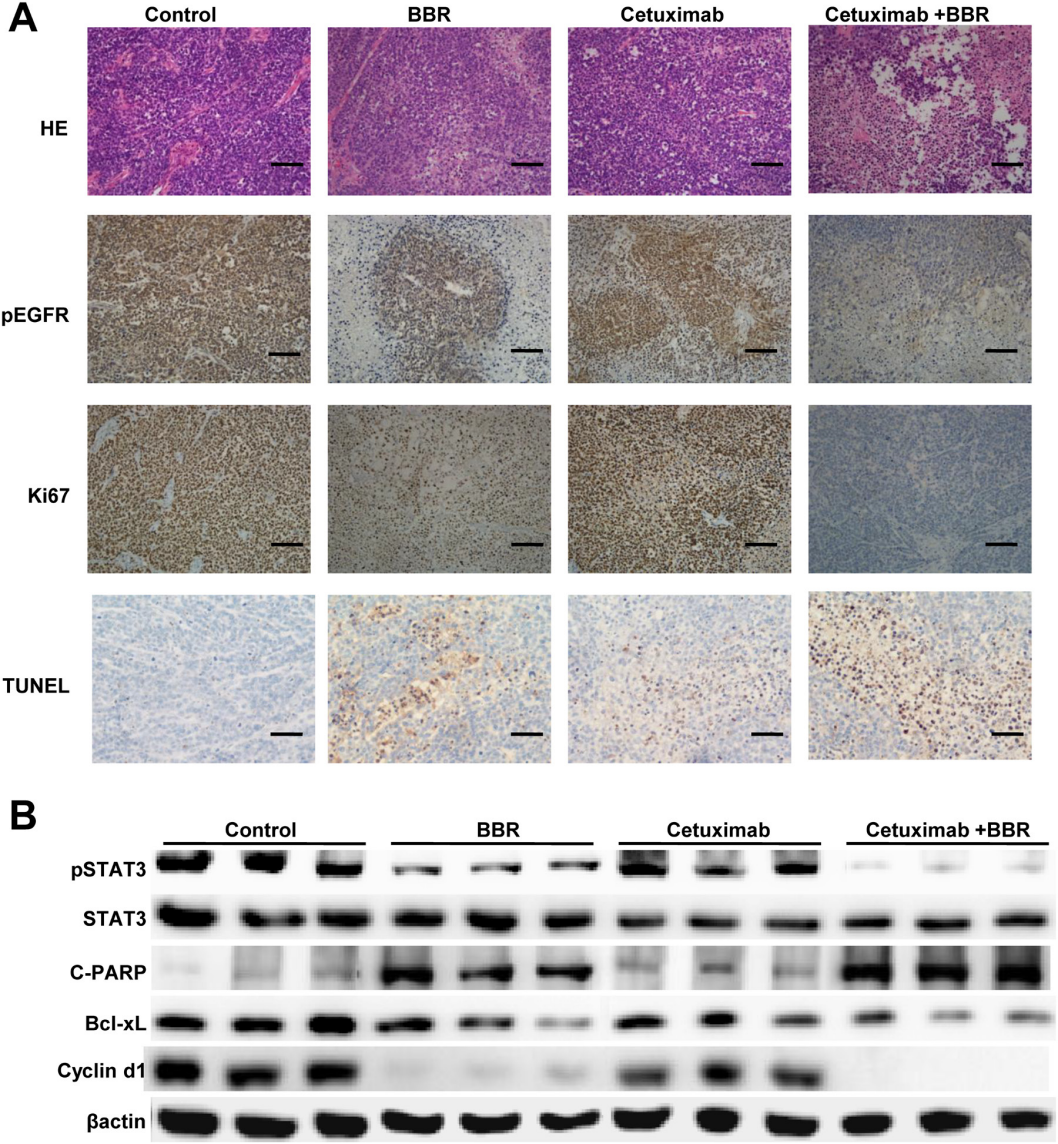
Berberine induces EGFR phosphorylation down-regulation, inhibits proliferation and enhanced the apoptosis in xenograft models **A.** Paraffin-embedded sections (4 μm) from tumor tissues were stained for HE, pEGFR, Ki67 and TUNEL using immunohistochemistry. Scale bars, 30 μm. **B.** Lysates were extracted from tumors of mice. Selected lysates, probed by immunoblot for pSTAT3, STAT3, C-PARP, Bcl-xL and cyclin D1, are shown, and which have been normalized to β-actin.

To validate why combination of berberine and cetuximab is more effective than either drug alone, we next analyzed EGFR/STAT3signaling pathway in xenografts. Immunohistochemistry showed high expression of p-EGFR and Ki67, low apoptotic bodies in the control. Berberine treatment alone or in combination with cetuximab increased apoptotic bodies and decreased p-EGFR and Ki67 expression (Figure 6A). Western blotting showed that decreased levels of tyrosine phosphorylated STAT3, Bcl-Xl and cyclin D1 in mice treated with cetuximab plus berberine compared to the control and cetuximab alone (Figures 6B).

### Berberine suppresses phosphorylation of receptor tyrosine kinases in GC cell lines

**Figure 7:**
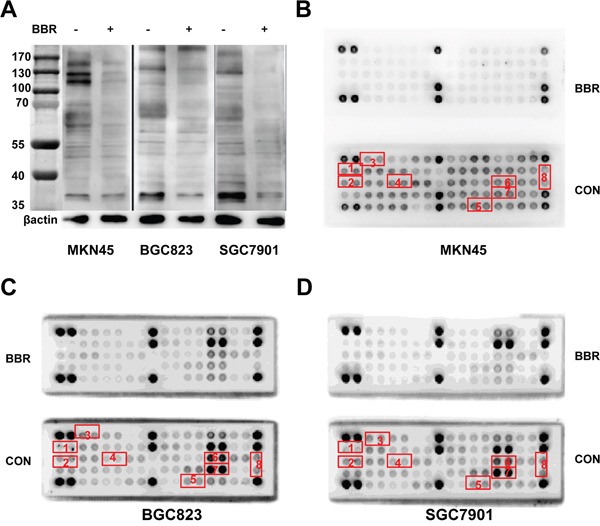
Berberine inhibits phosphorylation of receptor tyrosine kinases in GC cell lines **A.** Western blot analysis of extracts from MKN45, BGC823 and SGC7901 cells, untreated (−) or treated with berberine(BBR)(IC50, 24h), using Phospho-Tyrosine (P-Tyr-1000) MultiMab™ Rabbit mAb mix, β-actin was used as a loading control. **B, C** and **D.** Treatment of MKN45, BGC823 and SGC7901 cells with berberine inhibited phosphorylation of EGFR, HER2, FGFR1, c-MET, p44/42 MAPK and STAT3 as detected by the PathScan® RTK Signaling Antibody Array Kit (Chemiluminescent Readout). Cells were treated berberine (IC50) for 24 hours. Images were analyzed by ImageJ (http://rsbweb.nih.gov/ij/) CON: control, BBR: berberine, 1: p-EGFR, 2:p-HER2, 3:p-FGFR1, 4:p-MET, 5:p-VEGFR2, 6:p-ERK, 7:p-SRC, 8:p-STAT3.

We revealed that berberine inhibited EGFR signaling pathway in gastric cancer. To correlate the berberine to other tyrosine kinases activity, Phospho-Tyrosine (P-Tyr-1000) MultiMab™ Rabbit mAb mix and PathScan RTK Signaling Antibody Array kit were used (Figure 7). MKN45, BGC823 and SGC7901 cells were stimulated with berberine, or were treated with DMSO. After normalization to the negative control, we found that most of tyrosine kinase obtained lower phosphorylation signal in berberine group than DMSO group such as EGFR, ERBB2, FGFR1, MET, VEGFR2. These tyrosine antibody data suggest a possible signaling pathway regulated through the cell growth factor receptors pathway. Simultaneously, phosphorylation signals of ERK, SRC and STAT3 also reduced.

## DISCUSSION

EGFR is overexpressed in gastric cancers [16]. Phase 2 studies assessed the efficacy of cetuximab combined with chemotherapy for advanced gastric cancer. The results is active and drug toxicity is acceptable [17, 18]. However, in phase 3 trials additions of cetuximab to capecitabine-cisplatin don't provide additional benefit to chemotherapy alone in advanced gastric cancer [19]. Thus, novel strategies are an urgent need to enhance the effects of EGFR-TKIs in GC. In the current study, we investigated berberine's potential utility in the treatment of GC. Berberine was found to inhibit growth and induce apoptosis. Simultaneously, berberine effectively improved the antitumor effects of erlotinib and cetuximab on GC cell lines *in vitro* and *in vivo*.

It has been reported that berberine could suppress the constitutive activation of EGFR in human colon tumor, prostate cancer and glioblastoma [13, 14, 15]. Berberine has been proven to inhibit EGFR through activation of Cbl in colon tumor cells [13]. More recently, berberine downregulate the EGFR-MEK-ERK signaling pathway in human glioblastoma cells [15]. Furthermore, EGFR was inhibited in berberine treatment of prostate cancer cell lines [14]. In our study, berberine was found to decrease phosphorylation levels of EGFR both *in vitro* and *in vivo*. Berberine resulted in decreased levels of pERK, pAKT, pNFκB and pSTAT3, as well, suggesting global inhibition of the EGFR signaling pathway.

The dose of berberine we used *in vivo* experiments is just the same as used in gastroenteritis patients. Rats treated with berberine at a dose of 156 mg/kg/day for 90 days show no abnormality in clinical signs, body weights, organ weights, urinalysis, hematological parameters, gross necropsy and histopathology [20]. And in our experiment, mice received only dose of 50 mg/kg at daily diet. During the experiments, no obvious weight loss was observed in mice treated with berberine, cetuximab, or both. By Reagan–Shaw method [21], The human equivalent of the murine dose of 50 mg/kg is 250 mg in an adult of 60kg. The standard dose of berberine is 900-2,000 mg a day. Thus, the dose of breberine in our study is within a therapeutic range in humans.

Interestingly, EGFR-TKIs treatment resulted in AKT and NFκB activation in detected GC cell lines and maintenance activation of PI3K/AKT pathway signaling was associated with therapeutic resistance to EGFR-TKIs. It has been verified that AKT signaling is associated with the berberine treatment of GC cells [22]. So we silenced STAT3 and inhibited phosphorylation of ERK in BGC823 cells. Silencing STAT3 decreased Bcl-xL, cyclinD1 and increased cleaved PARP. However, ERK inactivation did not show similar results. Thus, our data provided evidence that berberine enhanced the antitumor effects of EGFR-TKI therapies by inhibiting EGFR/STAT3 signaling pathways.

It was reported recently that amplification of MET, FGFR1 and ERBB2 was involved in EGFR therapeutic resistance in colorectal cancer [23]. If these findings can be translated into gastric cancer settings, patients with high expression of MET, FGFR1 and ERBB2 may exhibit EGFR therapeutic resistance. Tissue microarray analyzed that HER2, EGFR, MET and FGFR2 predominances were respectively observed in 10.1, 13.9, 16.1 and 22.9% of the gastric adenocarcinomas. Nearly two-thirds of GC patients exhibited at least one RTK. Moreover, one-third of cases showed multiple RTKs expressions [24]. Thus, resistance of EGFR targeting therapies may be due to activation of alternative signaling pathways, including ERBB2, cMET or FGFR receptors. In this study, RTK Signaling antibody array showed that berberine could reduce phosphorylation levels of EGFR, ERBB2, FGFR1, cMET, VEGFR2 at the same time. Our results suggest berberine may potentially be useful in overcoming the resistance to EGFR targeting agents.

In addition, cetuximab combined with chemotherapy shows a serious side effect diarrhea [25]. Fortunately, berberine has become therapeutics for the treatment of diarrhea and gastroenteritis. It is used in the treatment of diarrhea of different origins [26, 27]. Accordingly, berberine combined with EGFR inhibitors to GC patients may be more effective and safer, because this combination treatment may not only increase EGFR-TKIs efficacy, but also prevent or alleviate the diarrhea symptom.

Therefore, administration of a compound that enhances the activity of GC treatment may be a useful complementary strategy. In this study, we have proven that berberine enhanced the activity of erlotinib and cetuximab *in vitro* and *in vivo* by inhibiting the EGFR/STAT3 signaling pathway, which suggests a potential role for berberine in the treatment of GC, particularly in combination with EGFR-TKIs therapy.

## MATERIALS AND METHODS

### Reagents and cells

Gastric cancer cell linesMKN45, BGC823, SGC7901, were maintained in RPMI-1640 medium (HyClone, Logan, UT, USA) with 10% fetal bovine serum (FBS) at 37°C in a humidified air atmosphere containing 5% CO2. Berberine chloride hydrate, 3-(4,5-dimethylthiazol-2-yl)-2,5-diphenyltetrazolium bromide (MTT) were purchased from Sigma-Aldrich Co. (St. Louis, MO, USA). Antibodies specific to EGFR, pEGFR, ERK, pERK (Thr202/Tyr204), AKT, pAKT, STAT3, pSTAT3, NFκB, pNFκB, Bcl-xL, Cleaved PARP, Cyclin D1 and Phospho-Tyrosine (P-Tyr-1000) MultiMab™ Rabbit mAb mix were purchased from Cell Signaling Technology Inc. (Beverly, MA). Antibody against actin was purchased from Santa Cruz Biotechnology (Santa Cruz, CA). Erlotinib and U0126(ERK inhibitor) was purchased from Cell Signaling Technology Inc. (Beverly, MA). Cetuximab was purchased from Merck Lyon Pharmaceutical Company Limited (GER).

### PathScan RTK antibody array kit

The PathScan RTK signaling array kit(Cell Signaling Technologies) contains fixed antibodies against phosphorylated forms of kinases and key signaling proteins. The kit includes 28 receptor tyrosine kinases and 11 important signaling nodes. It was used according to the manufacturer's instructions. Images were analyzed by ImageJ (http://rsbweb.nih.gov/ij/)

### Cell viability assay

Cell viability was detected using the MTT assay. Cells were cultured in triplicate in 96-well plates and treated with increasing concentrations of with berberine, erlotinib, cetuximab or the corresponding vehicles. After 48 hours, cell growth was measured by 0.5 mg/ml MTT. Cell viability was expressed as a percentage of control. The IC50 was calculated using Prism software version 4.03 (GraphPad Software Inc).

### Calculation of synergism

The medium-effect method was used to evaluate dose-response data for multiple drugs. The Chou and Talalay [28] combination index (CI), a well-established index reflecting the interaction of two drugs, was calculated at different levels of growth inhibition with the use of software package Calcusyn (Biosoft, Cambridge, UK). The CI for 50% growth inhibition (IC50) was calculated as follows:

CI values of <1, 1, and >1 indicate synergistic, additive, and antagonistic effects, respectively.

### Apoptosis assay

Cell apoptosis was assessed using an Annexin-V-FITC apoptosis detection kit (BD, Franklin Lakes, NJ, USA) as previously described [29].

### Western blotting

Following treatment with specific drugs, total cell lysates are prepared and subjected to SDS-PAGE using 8%, 10% or 12% running gels. Western blotting was done as previously described [30].

### *In vivo* tumor xenograft study

Female five-week-old BALB/C-nu/nu nude mice are obtained from the Shanghai Laboratory. The animal study protocols were approved by institutional animal care and use committee. BGC823 cells (2×10^6^ per mouse) were subcutaneously injected into the flank of each mouse. After outgrowth of palpable tumors, mice were randomized, by tumor volume, to 4 treatment groups. The cetuximab treatment group received 0.8 mg/mouse/day, by intraperitoneal injection, twice per week. The combination treatment group received both cetuximab, twice per week, and berberine. Berberine was dissolved in carboxymethylcellulose sodium for use in oral gavage (50 mg/kg body weight) daily. Tumors were measured using digital calipers twice per week and tumor volumes calculated using the following formula: Volume= L × (W)^2^/2 (L: longest diameter; W: shorter diameter). At the end of the study, mice were euthanized and tumor tissues harvested and frozen for analysis.

### Immunohistochemistry

The immunohistochemical staining of p-EGFR, Ki67 and TUNEL was performed as previously described [31]. Target protein expression was evaluated according to the ratio of positive cells per specimen, and staining intensity was determined according to a histological scoring method.

### Statistical analyses

The data were presented as the means ± standard errors of the mean (SEM) or medians (ranges). Student's t-test (two-tailed) or an one-way ANOVA test was employed to analyze the *in vitro* and *in vivo* data unless otherwise indicated (χ2 test). The non-parametric Mann-Whitney test was used to analyze the relationship between p-EGFR expression levels and various clinicopathologic characteristics. Statistical tests were performed using SPSS 17.0 software (Chicago, IL, USA). *P* < 0.05 was defined as statistically significant.
